# Identification of S-Nitrosylated (SNO) Proteins in *Entamoeba histolytica* Adapted to Nitrosative Stress: Insights into the Role of SNO Actin and *In vitro* Virulence

**DOI:** 10.3389/fcimb.2017.00192

**Published:** 2017-05-23

**Authors:** Meirav Trebicz-Geffen, Preeti Shahi, Shruti Nagaraja, Shai Vanunu, Shiran Manor, Amit Avrahami, Serge Ankri

**Affiliations:** ^1^Department of Molecular Microbiology, Ruth and Bruce Rappaport Faculty of Medicine, TechnionHaifa, Israel; ^2^Ruth and Bruce Rappaport Faculty of Medicine, TechnionHaifa, Israel

**Keywords:** *Entamoeba histolytica*, parasites, nitric oxide, F-actin, virulence

## Abstract

We have recently reported that *Entamoeba histolytica* trophozoites can adapt to toxic levels of the nitric oxide (NO) donor, S-nitrosoglutathione (GSNO). Even if the consequences of this adaptation on the modulation of gene expression in NO-adapted trophozoites (NAT) have been previously explored, insight on S-nitrosylated (SNO) proteins in NAT is missing. Our study aims to fill this knowledge gap by performing a screening of SNO proteins in NAT. Employing SNO resin-assisted capture (RAC), we identified 242 putative SNO proteins with key functions in calcium binding, enzyme modulation, redox homeostasis, and actin cytoskeleton. Of the SNO proteins in NAT, proteins that are associated with actin family cytoskeleton protein are significantly enriched. Here we report that the formation of actin filaments (F-actin) is impaired in NAT. Consequently, the ability of NAT to ingest erythrocytes and their motility and their cytopathic activity are impaired. These phenotypes can be imitated by treating control parasite with cytochalasin D (CytD), a drug that binds to F-actin polymer and prevent polymerization of actin monomers. Removal of GSNO from the culture medium of NAT restored the sensitivity of the parasite to nitrosative stress (NS) and its ability to form F-actin formation and its virulence. These results establish the central role of NO in shaping the virulence of the parasite through its effect on F-actin formation and highlight the impressive ability of this parasite to adapt to NS.

## Introduction

Amebiasis is caused by the single-celled protozoan, *Entamoeba histolytica*. The disease is mostly prevalent in developing countries, and is one of the three most common causes of death from parasitic diseases (WHO, 1997). The parasite has two stages in its life cycle in the host: the infective cyst and the invasive trophozoite. About nine out of 10 people who are infected with *E. histolytica* are asymptomatic and in those individuals who develop symptoms, bloody diarrhea (amebic colitis) and liver abscess are the most common symptoms. Amebic colitis is characterized by acute inflammation of the intestine with the release of cytokines, such as tumor necrosis factor α (TNFα), interleukin 8 (IL-8), interferon gamma (IFN-γ), and interleukin β (IL-1β), and the generation of micromolar concentrations of reactive oxygen species (ROS) and reactive nitrogen species (RNS) from activated cells of the host's immune system (for a recent review see Begum et al., 2015). In the non-symptomatic phase of the infection, the parasite is exposed in the large intestine to nanomolar concentrations of nitric oxide (NO) that is produced in intestinal epithelial cells by constitutive NO synthase (NOS) (Kolios et al., 2004) and as an intermediate in denitrification by the intestinal microbiota (Vermeiren et al., 2009). In contrast, the parasite is exposed to NO in micromolar concentrations during colitis, and the parasite is killed due to S-nitrosylation of key metabolic enzymes and fragmenting of the endoplasmic reticulum (ER) (Santi-Rocca et al., 2012). We recently demonstrated that exposure of the parasite to non-lethal concentration of NO can induce the resistance of the parasite to cytotoxic concentration of NO (Shahi et al., 2016b). Transcriptomic analyses of NO-adapted trophozoites (NAT) have revealed an unexpected function of N-acetyl ornithine deacetylase (NAOD) in the adaptation of the parasite to NO. This function does not depend on NAOD's catalytic activity but is mediated by blunting the detrimental effect of glyceraldehyde 3-phosphate dehydrogenase (GAPDH) on *E. histolytica* exposed to NS (Shahi et al., 2016b). Transcriptomics analyses of NAT have also revealed that genes that are associated with actin family cytoskeleton protein are significantly upregulated. The actin-rich cytoskeleton is central to ameba motility which is modulated by various acting binding proteins, such as myosin II and ABP16 [for a recent review see (Aguilar-Rojas et al., 2016)]. Indeed, motility and actin cytoskeletal dynamics functions are directly linked to the pathogenicity of the parasite (Aguilar-Rojas et al., 2016). Despite these instructive data on the transcriptomics of NAT, information on the identity of nitrosylated proteins in NAT is lacking. Here, we inform on the biological relevance of S-nitrosylated (SNO) proteins in NAT using resin-assisted capture (RAC) coupled with mass spectrometry (MS). The result of this analysis revealed the low correlation, except for cytoskeletal proteins, between transcript level changes and SNO proteins in NAT. We also report that (a) some of the parasite's functions, such as erythrophagocytosis, motility, and virulence, become impaired in NAT during its adaptation to NO and (b) these functions are restored when NAT are no longer exposed to NO.

## Materials and methods

### Chemicals and reagents

GSNO was purchased from Sigma-Aldrich, St. Louis, MO, USA. CytD was purchased from Cayman Chemical Company, Ann Arbor, Michigan, USA.

### Microorganisms

*E. histolytica* trophozoites strain HM-1:IMSS were grown under axenic conditions in Diamond's TYI S-33 medium at 37°C. Trophozoites in the exponential phase of growth were used in all experiments. Trophozoites adapted to GSNO (120 μM) were prepared using a previously described protocol (Shahi et al., 2016b). Trophozoites exposed to an acute NS (TEANS) (1.5 10^5^ trophozoites/ml) were incubated for 60 min in Diamond's TYI S-33 medium at 37°C amended with 350 μM GSNO (final concentration). Trophozoites treated with CytD (1.5 10^5^ trophozoites/ml) were incubated for 12 h in Diamond's TYI S-33 medium at 37°C amended with 5 μM CytD (final concentration).

### SNO-RAC analysis of S-nitrosylated proteins

A total protein extract was prepared by lysing NAT (5 × 10^7^) in 1% Igepal CA-630 (Sigma-Aldrich, St. louis, Mo, USA) in phosphate buffer saline (PBS). S-nitrosylated proteins in the total protein extract were detected by SNO-RAC using a previously described protocol (Hertz et al., 2014a). Captured proteins were eluted with buffer (10 mM HEPES, 0.1 mM EDTA, 0.01 mM neocuproine, 0.1% SDS, 100 mM 2-mercaptoethanol) for 20 min at room temperature (RT), and the proteins in each eluent were resolved on a 12.5% SDS-PAGE gel. Each gel was then stained with Coomassie blue dye (Brilliant Blue G, Sigma-Aldrich, St. louis, Mo, USA) and each lane was submitted independently for mass spectrometric (MS) analysis.

For the detection of actin in SNO-proteins, an aliquot (10%) of the eluted proteins that were captured in absence or presence of ascorbate were resolved on a 10% SDS-PAGE in SDS-PAGE running buffer (25 mM Tris, 192 mM glycine, 0.1% SDS). Proteins were electrotransferred in protein transfer buffer (25 mM Tris, 192 mM glycine, 20% methanol, pH 8.3) to nitrocellulose membranes (Protran® BA83, Whatman). The blots were first blocked using 3% skim milk, and then probed with 1:1,000 monoclonal actin antibody (clone C4, MP Biomedicals, Solon, Ohio, USA) for 1 h at room temperature. The blots were incubated with 1:5,000 horseradish peroxidase conjugated secondary antibody (Jackson ImmunoResearch, Enco Diagnostics, Israel) for 1 h at RT, and then developed using enhanced chemiluminescence (SuperSignal West Pico Chemiluminescent Substrate, ThermoFisher Scientific, USA).

### In gel proteolysis and mass spectrometry analysis

The proteins in each gel slice were reduced with 2.8 mM DTT (60°C for 30 min), modified with 8.8 mM iodoacetamide in 100 mM ammonium bicarbonate (room temperature for 30 min in the dark), and digested overnight in 10% acetonitrile and 10 mM ammonium bicarbonate with modified trypsin (Promega, Biological industries, Israel) at 37°C.

The resulting tryptic peptides were resolved by reverse-phase chromatography on 0.075 × 200-mm fused silica capillaries (J & W Scientific, Folsom, CA, USA) packed with ReproSil-Pur reversed phase material (Dr. Maisch GmbH, Germany). The peptides were eluted with a linear 95-min gradient of 7–40% and 8 min at 95% acetonitrile with 0.1% formic acid in water at flow rates of 0.25 μl/min. MS was performed by an ion-trap mass spectrometer (Orbitrap, Thermo Fisher Scientific, USA) in a positive mode of operation using a repetitively full MS scan followed by collision-induced dissociation (CID) of the seven most dominant ions selected from the first MS scan. The MS data was analyzed using Proteome Discoverer software (version 1.3) which searches the Ameba section of the NCBI non-redundant database and the decoy databases [in order to determine the false discovery rate (FDR)] using the Sequest and the Mascot search engines.

### PANTHER classification system

The online PANTHER Version 11.0 (http://pantherdb.org/; Rist et al., 2007) was used in this study. SNO proteins in NAT were classified by using the “protein class” ontology setting, the pie chart option and the percent of gene hit against total # Protein Class hits setting. The statistical overrepresentation test was performed using the default setting, the annotation data set corresponding to PANTHER protein class and the Bonferroni correction for multiple testing options selected.

### Determination of *E. histolytica* motility

Trophozoite motility was determined using the Costar Transwell system (8-μm pore size polycarbonate membrane, 6.5-mm diameter, Corning Inc, Corning, NY, USA; Gilchrist et al., 2008). For this purpose, trophozoites were first washed three times in serum-free Diamond's TYI-S-33 medium, and then suspended in serum-free Diamond's TYI-S-33 medium. A 500-μl aliquot of the suspension (25 × 10^4^ trophozoites/ml) was loaded into a transwell insert, which was placed in each well of a 24-well culture plate which contained serum-free Diamond's TYI-S-33 medium (500 μl/well). The 24-well culture plate with the loaded inserts was placed in anaerobic bags (Mitsubishi Gas Chemical Company, Inc., Tokyo, Japan), and incubated for 3 h at 37°C. At the end of the incubation, the inserts and culture medium were removed from each well, and trophozoite migration was determined by counting the number of trophozoites that were attached to the bottom of each well.

### Erythrophagocytosis assay

Erythrophagocytosis was assayed using a previously described protocol (Mora-Galindo et al., 1997). Briefly, Human red blood cells (Hrbcs) (5 × 10^7^) and trophozoites (5 × 10^5^) were mixed and incubated for 10 min at 37°C. Phagocytosis was stopped by adding distilled water. The average number of erythrocytes inside the trophozoites was quantified using a calibration curve and by reading the absorbance at 397 nm after suspending a pellet of parasites in 90% formic acid.

### Measurement of cytopathic activity

The rate of destruction of cultured HeLa cell monolayers by trophozoites was determined using a previously described protocol (Bracha and Mirelman, 1984). Briefly, *E. histolytica* trophozoites (2.5 × 10^5^ or 10^5^/well) in serum-free Diamond's TYI-S-33 medium were incubated with HeLa cell monolayers in 24-well tissue culture plates at 37°C for 60 min. The incubation was stopped by placing the plates on ice and unattached trophozoites were removed by washing the plates with cold PBS. The HeLa cells that remained attached to the plates were stained with methylene blue (0.1% in 0.1 M borate buffer, pH 8.7). The dye was extracted from the stained cells by 0.1 M HCl, and color intensity of extracted dye was measured spectrophotometrically at OD_660_.

### Determination of protein synthesis by surface sensing of translation (SUnSET)

SUnSET was performed using a previously described protocol (Hertz et al., 2014b; Shahi et al., 2016a). Briefly, trophozoites (2 × 10^6^/ml) were incubated with 10 μg/ml puromycin (Sigma-Aldrich, St. louis, Mo, USA), a structural analog of tyrosyltRNA, for 20 min at 37°C. The trophozoites were lysed using 1% Igepal (Sigma) in PBS. Whole proteins were resolved on a 10% SDS-PAGE in SDS-PAGE running buffer. Proteins were electrotransferred in protein transfer buffer to nitrocellulose membranes. Loading equivalency was determined by immunoblotting using a 1:10,000 monoclonal α-tubulin antibody (DM1A clone, Sigma-Aldrich, St. louis, Mo, USA). Puromycin was detected by immunoblotting using a 1:5,000 monoclonal puromycin antibody (12D10 clone, Millipore). After incubation with the primary antibody, the blots were incubated with 1:5,000 secondary antibody for 1 h at RT (Jackson ImmunoResearch, Enco Diagnostics, Israel), and then developed using enhanced chemiluminescence (SuperSignal West Pico Chemiluminescent Substrate, ThermoFisher Scientific, USA). Protein quantification/synthesis was estimated from the intensity of the immunoreactive blots (densitometry) using Fiji software (Schindelin et al., 2012).

### Immunofluorescence microscopy

*E. histolytica* trophozoites (1.5 10^5^ trophozoites/ml) were suspended in complete Diamond's TYI S-33 medium at 37°C and transferred onto acetone-cleaned glass coverslips that were placed in the bottom of each well of a 24-well plate. Trophozoites were incubated for 15 min at 37°C in order to allow them to adhere to the coverslip surface. The attached trophozoites were fixed with pre-warmed (37°C) 3.7% paraformaldehyde (PFA) for 30 min at RT. After fixation, the attached trophozoites were permeabilized with 0.1% Triton X-100/PBS for 1 min at RT. The coverslips were washed three times with PBS and quenched with PBS containing 50 mM NH_4_Cl for 30 min at RT. The coverslips were then blocked with 1% bovine serum albumin (BSA) in PBS (BSA/PBS) for 30 min at RT. The samples were then probed with 1:500 monoclonal actin antibody (clone C4, MP Biomedicals, Solon, Ohio, USA) overnight. This monoclonal actin antibody was successfully used to detect *E. histolytica* actin (Perdomo et al., 2013) The next day, the samples were first washed three times in PBS, followed by two washes in 1% BSA/PBS, and then incubated with 1:250 Alexa Flour 488 (Jackson ImmunoResearch, PA, USA) and 1:1,000 4′,6-diamidino-2-phenylindole (DAPI) (MP Biomedicals, Solon, Ohio, USA) for 3 h at 4°C. At the end of the incubation, coverslips were incubated overnight at 4°C with 20 μM phalloidin [1 μM] conjugated to rhodamine (phalloidin conjugated to rhodamine was generously given by Prof. Adi Salzberg, Rappaport institute of Medicine, Technion, Haifa, Israel).

After incubation, the coverslips were washed three times in 1% BSA/PBS, and then with PBS. The samples were then mounted onto microscope slides with Fluoromount G (SouthernBiotech, Birmingham, AL, USA). The specimens were then examined under a confocal immunofluorescence microscope (ZEISS- LSM510 Meta Laser Scanning System confocal imaging system) with a 63X oil immersion objective.

Fluorescent quantification of F-actin in control trophozoites, NAT, NAT that have been cultivated for 1 month in absence of GSNO (NATR) and trophozoites treated with CytD was performed using Fiji software (Schindelin et al., 2012).

### Statistical analysis

Data are presented as the mean ± standard deviation (SD). Significant differences between two groups were determined using an unpaired Student's *t*-test with a significance level of 0.05.

## Results

### SNO-RAC analysis of S-nitrosylated proteins in NAT

**Figure 1:**
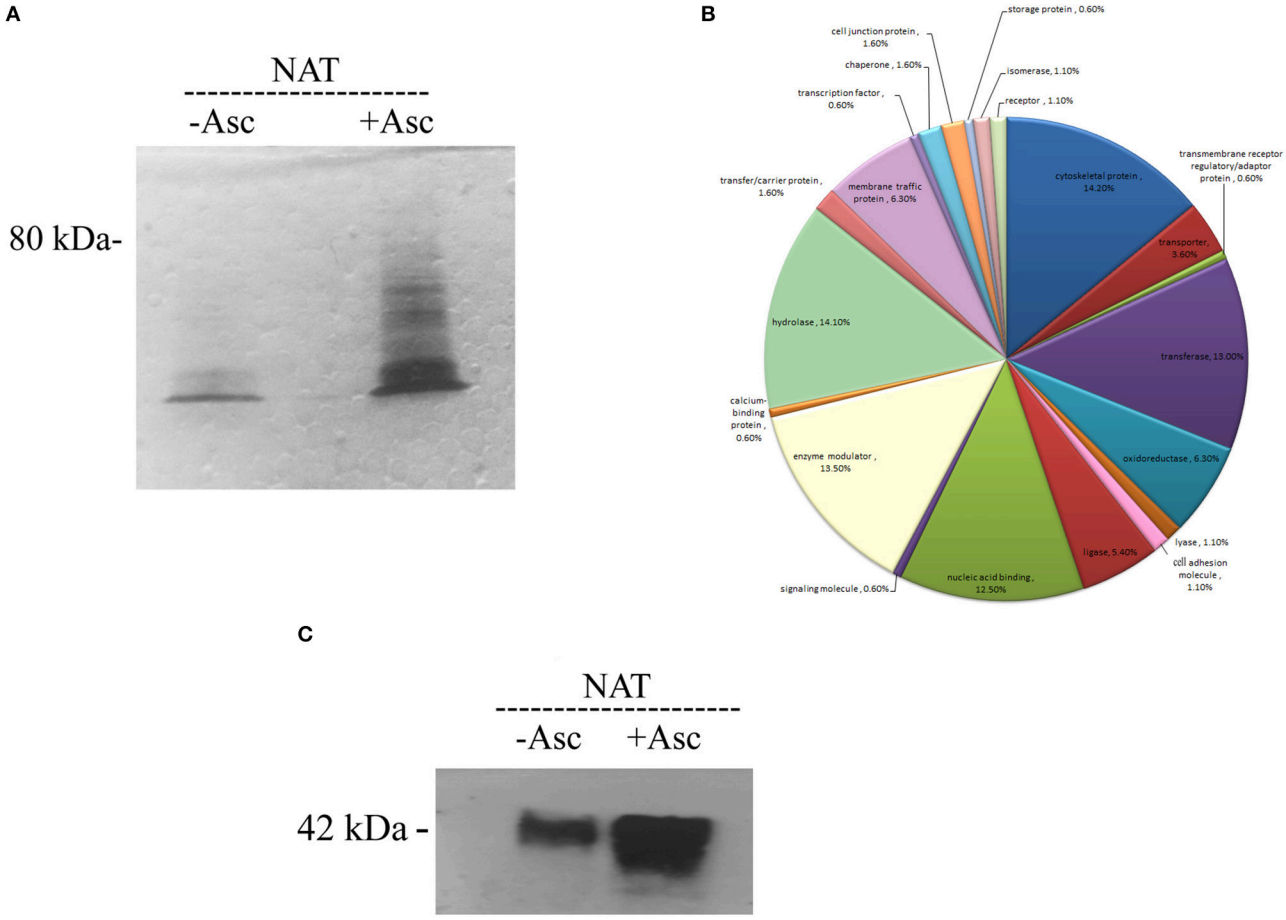
**Analysis of SNO proteins in NAT after resin-assisted capture. (A)** Coomassie blue staining of SNO proteins. SNO proteins in whole protein lysate of NAT were subjected to RAC in the presence of 40 mM ascorbate (+ASC) or the absence of ascorbate (–ASC). **(B)** Functional categories of all SNO proteins. SNO proteins in NAT were classified according to their biological role. **(C)** Confirmation of S-nitrosylation of actin after RAC by western blotting. This figure displays a representative result from two independent experiments.

The amounts of SNO proteins in the untreated and ascorbate-treated (40 mM) total protein extract of NAT was determined by SNO-RAC coupled to MS (Hertz et al., 2014a; Figure 1A). A protein was considered to be a SNO protein when this protein was present in the ascorbate-treated lysates of at least two independent assays and not in the untreated lysates. From the results of three independent analyses, we found that 242 proteins fulfilled this criterion (Supplementary Table 1 and for more details Supplementary Tables 2, 3). These proteins included cytoskeletal protein (such as actin (EHI_159150) or paxillin (EHI_050720) (Table 1), signal transduction (such as GTP binding protein (EHI_148270) or RNA GTPase (EHI_148190), hydrolase (such as plasma membrane calcium transporting ATPase (EHI_016480), ligase [such as AcetylCoA synthase (EHI_135740) or long chain fatty acid-CoA ligase (EHI_153720)], Nucleic acid binding (this category is mostly represented by ribosomal proteins (RP) like 60 RPL2 (EHI_183480) or 40S RPS3a (EHI_065270), oxidoreductase (such as dihydropyrimidine dehydrogenase DPD (EHI_012980) or alcohol dehydrogenase (ADH) (EHI_198760) and transferase such as S-adenosyl-methionine synthetase (EHI_004920) and sulfate adenylyltransferase (EHI_197160) (Figure 1B). In order to confirm the consistency of our SNO-RAC analysis, actin was selected and the presence of SNO actin was confirmed by western blotting (Figure 1C). We observed that the amounts of actin which bound to the thiopropyl sepharose beads were significantly smaller in the untreated samples than the amounts in the ascorbate-treated samples. This result indicates that the binding of actin to the thiopropyl sepharose beads was due to its S-nitrosylation and not due to the background binding of the protein to the beads (Figure 1C). The results of the MS analysis of actin that was bound to the thiopropyl sepharose resin in the presence of sodium ascorbate revealed the presence of carbamidomethylated cysteine residues at positions 18, 218, and 286 (Supplementary Table 1). These residues possibly correspond to S-nitrosylated cysteines that have been reduced by the ascorbate, bound to the resin, eluted by 2- mercaptoethanol, and alkylated by iodoacetamide prior to digestion of the protein and MS analysis.

**Table 1:** **Cytoskeletal proteins identified by the PANTHER classification system**.

**Protein name**	**Gene symbol**
Actin	EHI_107290
Actin	EHI_107290
Putative uncharacterized protein	EHI_068510
Actin-binding protein, cofilin/tropomyosin family	EHI_168340
Dynamin-like protein	EHI_052740
Putative uncharacterized protein	EHI_169670
Myosin heavy chain	EHI_140720
Orphan actin related protein (ARPvii)	EHI_008780
Actin	EHI_107290
Tubulin alpha chain	EHI_005950
Plastin-2, putative	EHI_105670
Putative uncharacterized protein	EHI_118750
Putative uncharacterized protein	EHI_021360
Paxillin, putative	EHI_050720
LIM zinc finger domain containing protein	EHI_158150
Coronin	EHI_082080
Actin	EHI_107290
Filamin 2	EHI_104630
Actin	EHI_107290
Myosin heavy chain	EHI_110180
Putative uncharacterized protein	EHI_065490
Filopodin, putative	EHI_080740
Actin	EHI_107290
Uncharacterized protein	EHI_148050
Coronin	EHI_083590
Actin	EHI_107290
ARP2/3 complex 20 kDa subunit	EHI_030820
ARP2/3 complex 20 kDa subunit	EHI_030820

According to the results of the PANTHER statistical overrepresentation test, actin and actin related proteins, such as actin (EHI_159150) or orphan actin related protein (ARPvii; EHI_008780; fold enrichment 13 and *P*-value 3.53E^−05^) and oxidoreductase, such as DPD (EHI_012980) or ADH (EHI_198760; fold enrichment 5.2 and *P*-value 1.53E^−03^), were significantly enriched.

### Comparison between SNO proteins in nat and in TEANS

We found 27 common SNO proteins in NAT and in TEANS (Hertz et al., 2014a). According to the results of PANTHER statistical overrepresentation test, those with oxidoreductase activity and structural constituent of ribosome are the most enriched among these 27 SNO proteins (Table 2).

**Table 2:** **Common SNO proteins in NAT and in TEANS**.

**Protein name**	**Gene symbol**
Galactokinase, putative	EHI_094100
Hemolysin-3, putative	EHI_080730
Type A flavoprotein, putative	EHI_096710
NAD(P) transhydrogenase subunit alpha, putative	EHI_014030
Alcohol dehydrogenase, putative	EHI_198760
Phosphoribulokinase /uridine kinase family protein	EHI_087540
40S ribosomal protein S4, putative	EHI_118170
2,3-bisphosphoglycerate-independent phosphoglycerate mutase, putative	EHI_050940
Coronin	EHI_083590
60S ribosomal protein L18a	EHI_035600
Glycyl-tRNA synthetase, putative	EHI_073460
ARP2/3 complex 20 kDa subunit, putative	EHI_030820
3′(2′),5′-bisphosphate nucleotidase, putative	EHI_193350
Putative uncharacterized protein	EHI_029350
Putative uncharacterized protein	EHI_140360
Enolase, putative	EHI_130700
Malate dehydrogenase, putative	EHI_092450
LIM zinc finger domain containing protein	EHI_158150
Coatomer subunit gamma	EHI_040700
60S ribosomal protein L10, putative	EHI_044810
Dihydropyrimidine dehydrogenase, putative	EHI_012980
Hypothetical protein	EHI_030750
Acetyl-CoA synthetase, putative	EHI_178960
Gal/GalNAc lectin heavy subunit	EHI_012270
Hypothetical protein	EHI_178470
Rubrerythrin	EHI_134810
Guanine nucleotide-binding protein alpha-16 subunit	EHI_140350

### Comparison between gene expression and SNO proteins in NAT

Only seven genes have both their expression differentially regulated in NAT (Shahi et al., 2016b) and have their product nitrosylated. These genes are plasma membrane calcium-transporting ATPase, (EHI_016480), 26s protease regulatory subunit (EHI_052050), phosphorylase (EHI_096830), myosin heavy chain (EHI_110180), helicase (EHI_148930), uncharacterized protein (EHI_189410), and a putative D-3-phosphoglycerate dehydrogenase (EHI_060860). According to the results of the PANTHER statistical overrepresentation test, the actin family of cytoskeletal proteins are significantly enriched among the products of upregulated genes in NAT (Shahi et al., 2016b) and SNO proteins in NAT (this work).

### Proteins synthesis in NAT

**Figure 2:**
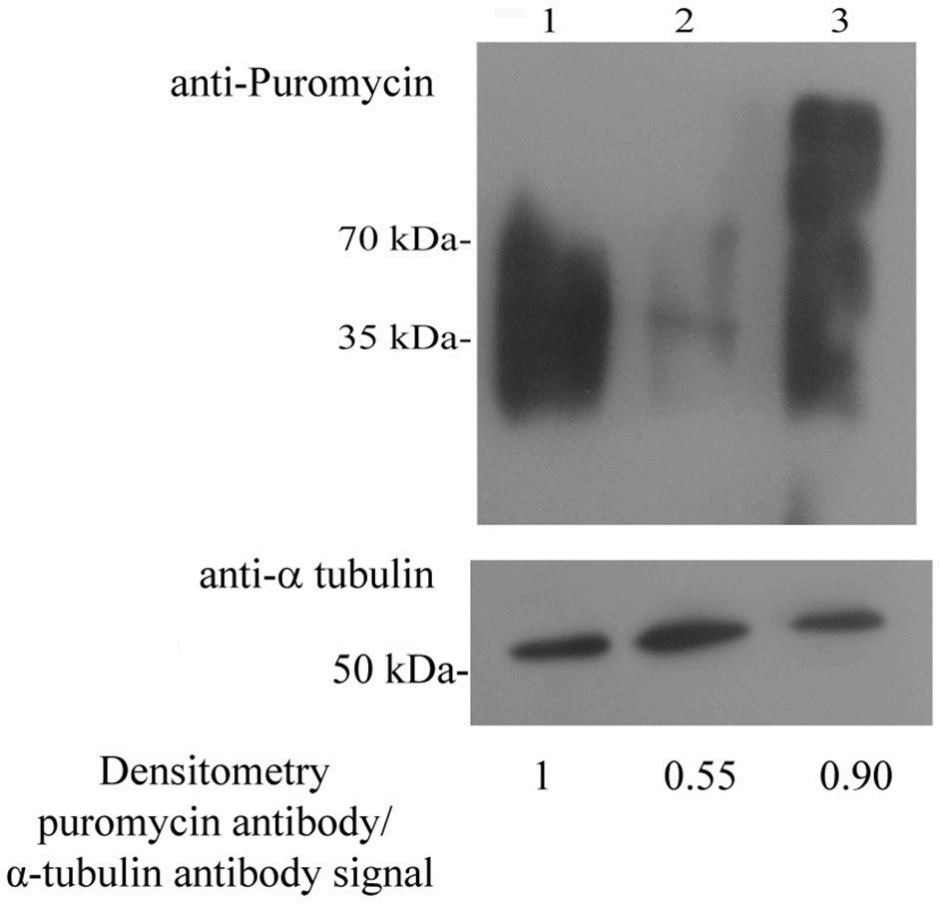
**Protein synthesis, measured using puromycin-labeled proteins in control ***E. histolytica*** trophozoites, TEANS, and NAT Lane 1**. Control *E. histolytica* trophozoites. Lane 2. TEANS. Lane 3. NAT. Whole protein extracts were separated by denaturing electrophoresis and analyzed by western blotting **(upper panel)** using a puromycin antibody or a α-tubulin antibody **(lower panel)**. Densitometric quantification of the puromycin antibody/α-tubulin antibody signal. The signal from control trophozoites was set at 1 and the results are representative of two independent experiments.

We previously reported that an acute NS inhibits protein synthesis in *E. histolytica* (Hertz et al., 2014a). The presence of proteins which are involved in translation among the SNO proteins in NAT, such as 60 RPL2 and 40S RPS3a, suggests that NO regulates the translation of proteins in NAT. In order to test this hypothesis, we used the SUnSET (Schmidt et al., 2009), to determine the amount of puromycin that was incorporated into nascent peptide chains (Figure 2). As previously described (Hertz et al., 2014b), we found that protein synthesis is strongly inhibited in TEANS compared to that in control (untreated) trophozoites. In contrast, we found that protein synthesis in NAT is comparable to that in control trophozoites (Figure 2).

### Effect of S-nitrosylation on the actin cytoskeleton

**Figure 3:**
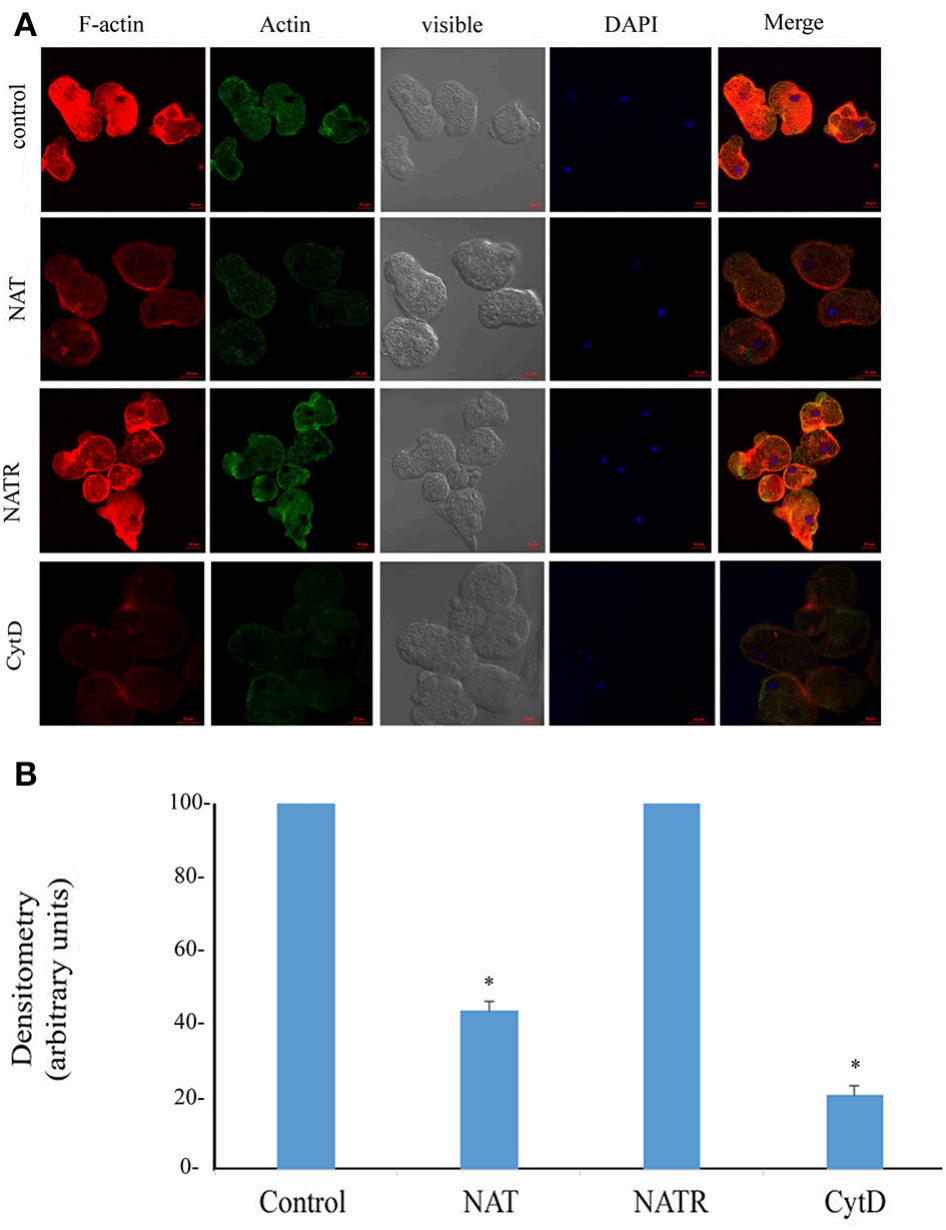
**(A)** Confocal laser scanning microscopy of F-actin and total actin in control *E. histolytica* trophozoites, NAT, NATR, and CytD-treated *E. histolytica* trophozoites. F-actin was detected using rhodamine-conjugated phalloidin. Total actin was detected using a primary actin antibody and a secondary Cy2-conjugated IgG antibody. The nuclei (blue) were stained by DAPI. Computer-assisted image overlay of the signal emitted by the actin antibody, phalloidin, and DAPI. **(B)** Analysis of the F-actin signal (fluorescence) in control trophozoites, NAT, NATR and CytD-treated trophozoites. The analysis has been performed with the Fiji software on 20 trophozoites. The signal from control trophozoites was set at 100 and data are displayed as the mean ± standard deviation. ^*^p < 0.05.

**Figure 4:**
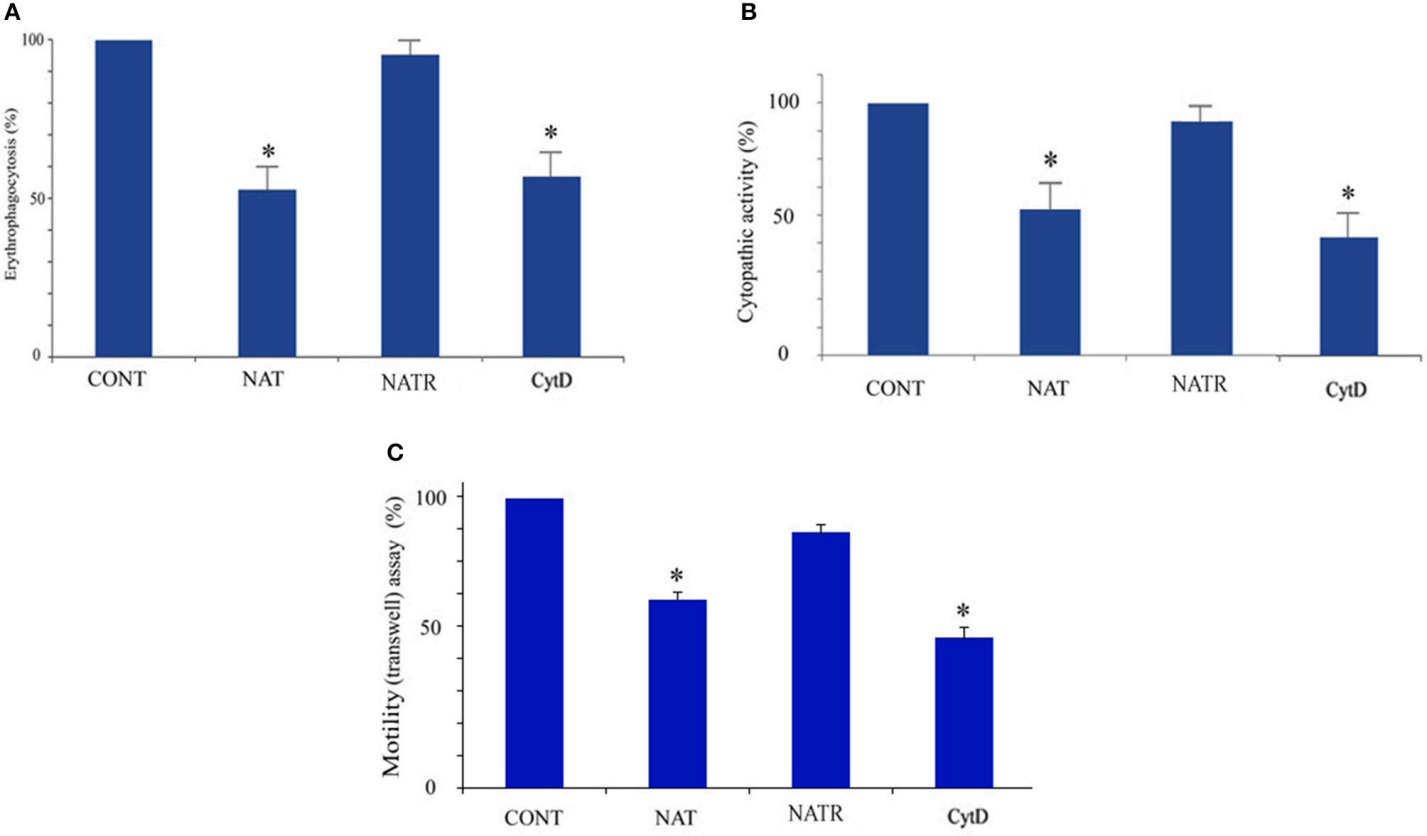
**Determination of cytoskeleton-dependent virulence markers in control ***E. histolytica*** trophozoites, NAT, NATR, and CytD-treated ***E. histolytica*** trophozoites Erythrophagocytosis (A)** cytopathic activity **(B)** and motility **(C)** of control *E. histolytica* trophozoites, NAT, NATR, and CytD-treated *E. histolytica* trophozoites were examined. The value from control *E. histolytica* trophozoites for each activity was set at 100%. Data are displayed as the mean ± standard deviation of three independent experiments that were repeated twice with a *P*-value, 0.05. ^*^p < 0.05.

The enrichment of actin family of cytoskeletal proteins among SNO proteins and among the products of genes that are upregulated in NAT (Shahi et al., 2016b) motivated us to investigate the presence of F-actin in control trophozoites and NAT by immunofluorescence microscopy with phalloidin, a molecule which selectively binds to F-actin. We found that the intensity of the F-actin signal in NAT is one third of that in control trophozoites (Figures 3A,B). In contrast, we previously found that the intensity of the NAOD signal or the GAPDH signal in NAT is the same of that in control trophozoites (Shahi et al., 2016b). NAOD or GAPDH are not included among SNO-proteins in NAT (Supplementary Table 1). CytD inhibits actin polymerization by binding to F-actin (May et al., 1998). We found that the same amount of F-actin was found in CytD-treated control trophozoites and NAT (Figures 3A,B). The finding of a reduced amount of F-actin in NAT and CytD-treated trophozoites was confirmed by immunofluorescence microscopy with an actin antibody (Figures 3A,B). Attempts to study the amount of F-actin in CytD-treated NAT were unsuccessful due to the high toxicity of CytD to NAT (data not shown). These results strongly suggest that GSNO impairs the formation of F-actin in NAT. Actin polymerization is essential for the phagocytic and cytopathic activities of *E. histolytica* (Godbold and Mann, 1998). A comparison between control trophozoites and NAT was carried out using the erythrophagocytosis assay (Figure 4A). The extent of erythrophagocytosis by NAT was half of that of the control trophozoites. We also found that erythrophagocytosis was impaired in CytD-treated control trophozoites (Figure 4A). Cytopathic activity of NAT was less than that of control trophozoites. Furthermore, CytD impairs the cytopathic activity of control trophozoites (Figure 4B). Since actin polymerization is an essential process during migration of the parasite (Emmanuel et al., 2015), we compared the migration of control trophozoites and NAT using the transwell migration assay. We found that the number of control trophozoites that passed through the pores was considerably greater than the number of NAT (Figure 4C). Furthermore, treatment of control trophozoites with CytD impairs their migration (Figure 4C). Collectively, these results indicate that virulence markers that depend on F-actin formation are impaired in NAT.

### Reversibility of NAT phenotypes

The fact that S-nitrosylation is a redox-reversible posttranslational protein modification (Gould et al., 2013) motivated us to determine if NAT phenotypes are reversible.

We previously reported that adaptation of *E. histolytica* to NO has a negative effect on its generation time (Shahi et al., 2016b). The generation time of NAT was half of that of control trophozoites (20 ± 1 h vs. 10.2 ± 0.5 h; *p* < 0.005). We also found that the generation time of NATR and control trophozoites are similar (10.3 ± 0.5 h). NAT can be continuously cultivated in presence of 110 μM GSNO (Shahi et al., 2016b). In contrast, NATR, like control trophozoites, died within 48 h when exposed to GSNO (120 μM; data not shown). Erythrophagocytosis, cytopathic activity, motility and the level of F-actin of NATR were tested. We found that the virulence and the level of F-actin in NATR and control trophozoites are similar (Figures 3A,B, 4A–C).

## Discussion

*E. histolytica* has a remarkable ability to adapt to various stresses, such as glucose starvation (Baumel-Alterzon and Ankri, 2014), serum starvation (Ahamad et al., 2015), treatment with metronidazole (Penuliar et al., 2015), or NS (Shahi et al., 2016b) and comprehensive transcriptome analyses have been conducted in these stressed parasites. Redox proteomics (Hertz et al., 2014a; Shahi et al., 2016a) have provided new information and complementary results to the transcriptomics of *E. histolytica* trophozoites exposed to oxidative stress (OS) or NS (Santi-Rocca et al., 2012; Pearson et al., 2013). For example, the mRNA level of *E. histolytica* GalNAc lectin heavy subunit is not regulated by NO (Santi-Rocca et al., 2012) but the activity of this protein is inhibited by S-nitrosylated cysteines in the carbohydrate recognition domain (Hertz et al., 2014a). For this reason, we decided to extend the transcriptomics analysis of NAT by performing SNO-RAC and we identified 242 SNO proteins in NAT. Hundreds of SNO proteins were also identified in TEANS (Hertz et al., 2014a) which suggests that the mechanism of adaptation of NAT to NS is not based on the conversion of NO to harmless products (Vine and Cole, 2011). If such a mechanism exists, the number of SNO proteins that would be detected in NAT would be smaller than the number that would be detected in TEANS. The fact that we identified only 27 common SNO proteins in NAT and TEANS (Hertz et al., 2014a) suggests that the mechanism of adaptation to NS and the response of the parasite to acute NS is different. This assumption is also supported by the weak overlapping between genes expressed in NAT compared to genes expressed in TEANS (Santi-Rocca et al., 2012; Shahi et al., 2016b).

Nucleic acid binding proteins, such as the RPs, 60 RPL2, (EHI_183480) and 40S RPS3a (EHI_065270) are among the most represented group of SNO proteins that were identified in NAT. RPs are S-nitrosylated in TEANS (Hertz et al., 2014a) and oxidized in oxidatively stressed trophozoites (Shahi et al., 2016a) which leads to the inhibition of protein synthesis (Hertz et al., 2014b; Shahi et al., 2016a). Different mechanisms, such as NO- mediated cleavage of 28S and 18S rRNA (Cai et al., 2000) and NO-induced phosphorylation of eukaryotic initiation factor 2 (eIF-2) (Kim et al., 1998), have been proposed to explain this inhibition. This work shows that protein synthesis is not inhibited in NAT despite the presence of SNO-RPs. One possible reason to explain why protein synthesis is not inhibited in NAT is the replacement of SNO-RPs by newly synthesized RPs. Since the expression of SNO-RPs identified in this study was not upregulated in NAT (Shahi et al., 2016b), this proposed explanation cannot be accepted as valid explanation. Another explanation could involve the enzyme, glyceraldehyde 3-phosphate dehydrogenase (GAPDH). GAPDH is a glycolytic enzyme and a multitasking moonlighting protein (Jeffery, 2009). Among its moonlighting function, GAPDH binds to RPL13a and protects this RP against degradation (Jia et al., 2012). This protective function is lost when GAPDH is S-nitrosylated (Jia et al., 2012). We have recently showed that GAPDH is detrimental to *E. histolytica* exposed to NS (Shahi et al., 2016b). This toxic effect is reversed by the binding of N-acetylornithine deacetylase (NAOD) to GAPDH (Shahi et al., 2016b). We have also observed that ribosomal proteins, including RPL13, are co-purified with the NAOD-GAPDH complex (Shahi et al., 2016b). Therefore, it is tempting to speculate that NAOD prevents the formation of SNO-GAPDH thereby allowing GAPDH to prevent the degradation of RPs.

We identified S-adenosyl-methionine synthetase (SAM synthetase) (EHI_004920) as one of the SNO proteins in NAT. SAM synthetase catalyzes the formation of SAM from methionine and ATP. Inhibition of SAM synthetase by NO has been described for the rat enzyme (Perez-Mato et al., 1999). The inhibition is mediated by the S-nitrosylation of cysteine 90 which is essential for the activity of SAM synthetase (Reczkowski and Markham, 1995). Since cysteine 90 is conserved in *E. histolytica* SAM synthetase (data not shown), this finding suggests that the amebic enzyme is also inhibited by NO. If this is the case and in regards to the important biological processes that request SAM as methyl donor for the methylation of a large variety of substrates (DNA, proteins, lipids and many other small molecules) and polyamine synthesis (Mato et al., 2013), it is not surprising that NAT growth rate is affected (Shahi et al., 2016b).

We identified dihydropyrimidine dehydrogenase (DPD; EHI_012980) as one of the SNO oxidoreductases. We previously reported that DPD is essential for the adaptation of *E. histolytica* to glucose starvation (Baumel-Alterzon et al., 2013). The presence of DPD among the SNO proteins suggests that this enzyme is redox-regulated and that it may be involved in the adaptation to NO. However, overexpression of DPD does not provide any selective advantage to the parasite during its adaptation to NS (data not shown). The binding of pyrimidine to mammalian DPD is causing the closure of the active site loop that positions a catalytically crucial cysteine (C671) residue in the CSP motif (Lohkamp et al., 2010). Since this CSP motive is conserved in *E. histolytica* DPD, it is tempting to speculate that S-nitrosylation of this cysteine residue will inhibit the enzyme.

We identified NADP-ADH (EHI_023110) and ADH (EHI_198760) as SNO alcohol dehydrogenase in NAT. In contrast, five alcohol dehydrogenase including the bifunctional aldehyde-alcohol dehydrogenase 2 (ADH2) (EHI_150490) were identified in TEANS (Hertz et al., 2014a). ADH2 (EHI_150490) is essential for the parasite (Espinosa et al., 2001) and it catalyzes the formation of ethanol and the reoxidation of NADH (Pineda et al., 2010). S-nitrosylation of ADH2 caused its inhibition (Siman-Tov and Ankri, 2003; Santi-Rocca et al., 2012) and this inhibition may contribute to the amebicide effect of NO (Hertz et al., 2014a). Interestingly, SNO-ADH2 was not identified in NAT. This information suggests that during adaptation to NS, the parasite establishes a mechanism to protect ADH2 from S-nitrosylation. This mechanism may involve heat shock protein 60 (HSP60) (EHI_178570) which was identified as a SNO-protein in NAT. This hypothesis is supported by the fact that mouse HSP60 can protect the antioxidant enzyme manganese superoxide dismutase from inactivation by free radicals (Magnoni et al., 2014).

We found that cytoskeletal proteins, such as actin, are S-nitrosylated in NAT. *E. histolytica* relies on its dynamic actin cytoskeleton for invading the host's tissues (Aguilar-Rojas et al., 2016). S-nitrosylation or oxidation of the actin cytoskeleton can modulate its cellular functions in mammalian cells (Frenkel et al., 1996; Dalle-Donne et al., 2000; Fremont et al., 2017). S-nitrosylation of actin inhibits its polymerization and results in rearrangement of the cytoskeleton (Dalle-Donne et al., 2000; Rodriguez-Serrano et al., 2014). Actin's five cysteine residues (Cys217, Cys257, Cys272, Cys285, and Cys374) are highly vulnerable to redox modifications, and of these five residues, Cys374 is the most vulnerable oxidation, glutathionylation, carbonylation, and nitrosylation (Terman and Kashina, 2013). Modification of the Cys-272 and Cys-285 (Terman and Kashina, 2013) and probably the Cys374 (Dalle-Donne et al., 2007) residues has been linked to decreased actin polymerization and altered interactions with actin regulatory proteins. Actin is a highly conserved protein among species (Dominguez and Holmes, 2011) and cysteine residues identified as susceptible to S-nitrosylation are present in *E. histolytica* actin. The results of the MS analysis of actin that was bound to the thiopropyl sepharose resin in the presence of sodium ascorbate suggest that Cys-286 (the amebic equivalent of mammalian Cys-285) was S-nitrosylated. Since the parasite's virulence depends on an intact actin cytoskeleton (Lopez-Contreras et al., 2013), these results suggest that S-nitrosylation of actin inhibits those functions which are related to the parasite's virulence.

In this work we showed that fitness cost paid by the parasite to adapt to NO is an impairment of virulence functions that depends on a functional cytoskeleton. Although this price may seem excessive, it is paid off by the fact that upon relieve of the selection pressure, the parasite returns to its original level of virulence. To conclude, this investigation informs on the results of the s-nitrosoproteome of NAT. Although it is difficult to deduce from our data whether such SNO modifications actually occur when the parasite resides in its host, the results of this investigation highlight the important function of NO and the impact of S-nitrosylation of structural components of the cytoskeleton on *E. histolytica*'s virulence.

## Author contributions

SA conceived and designed this project and experiments. SA, MT, PS, SN, SV, SM, and AA performed the experiments. SA, MT, PS, SN, SV, SM, and AA analyzed the data and contributed to the development of the figures and tables. SA, MT, and PS wrote the manuscript. All authors reviewed the manuscript.

## Funding

This study was supported by the Israel Ministry of Health within the framework ERA-NET Infect-ERA (031L0004) (AMOEBAC project) and grants from the Israel Science Foundation (ISF) (260/16) and U.S.–Israel Binational Science Foundation (BSF) (2015211).

### Conflict of interest statement

The authors declare that the research was conducted in the absence of any commercial or financial relationships that could be construed as a potential conflict of interest.
